# Assessment of Knowledge and General Attitudes of Primary Care Physicians Toward Dipeptidyl Peptidase-4 (DPP-4) Inhibitor Drugs in the Management of Type 2 Diabetes Mellitus in the Qassim Region

**DOI:** 10.7759/cureus.86948

**Published:** 2025-06-29

**Authors:** Faisal Aljulajil, Unaib Rabbani

**Affiliations:** 1 Family Medicine Academy, Qassim Health Cluster, Buraydah, SAU

**Keywords:** diabetes mellitus, dpp-4 inhibitors, management, primary care, saudi arabia

## Abstract

Background: Dipeptidyl peptidase-4 (DPP-4) inhibitors play a significant role in type 2 diabetes mellitus (T2DM) management due to their efficacy and favorable safety profiles. Despite their growing importance, there is limited information on the knowledge and prescribing behaviors of primary care physicians in Saudi Arabia, particularly in the Qassim region, regarding the use of DPP-4 inhibitors. This study sought to assess the knowledge, attitudes, and prescribing patterns of primary care physicians concerning DPP-4 inhibitors in the management of T2DM.

Methods: A cross-sectional study was conducted involving 161 primary care physicians in the public sector from the Qassim region. Data were collected through a semi-structured and validated self-administered questionnaire that collected data on knowledge about DPP-4 inhibitors, attitudes toward their use, and prescription practices. Statistical analyses, both descriptive and inferential, were performed using the Statistical Package for the Social Sciences (SPSS) version 25 (IBM Corp., Armonk, NY, US).

Results: Participants showed specific knowledge gaps, but overall, most had a positive outlook. A significant majority (86.3%) were aware of the low risk of hypoglycemia associated with these agents; however, only 60.9% recognized them as a relatively new therapeutic option. Overall, physicians demonstrated positive attitudes and appropriate prescribing practices. Knowledge levels showed significant associations with physician age (<0.001), clinical experience (<0.001), qualifications (<0.001), and the availability of educational resources (<0.001).

Conclusion: Primary care physicians in the Qassim region generally possess good awareness and favorable attitudes toward DPP-4 inhibitors. However, further targeted educational programs are essential to bridge identified knowledge gaps and enhance prescribing practices for optimal T2DM care.

## Introduction

In today's society, diabetes mellitus, a chronic metabolic condition of glucose-insulin homeostasis, is on the rise. Around 589 million people were thought to have the condition globally as of 2024, with 85%-95% of those people having type 2 diabetes [1]. Furthermore, this number is expected to rise to 853 million by 2050 [1]. The Middle East and North African region is the third-largest region in terms of the number of people with diabetes. It is estimated that Saudi Arabia has about 5.3 million people with diabetes and an age-standardized prevalence of 23.1% [1].

Over the last few decades, diabetes has become one of the most common metabolic disorders in adults with demographic and epidemiological transition. Therefore, any physician in practice is unlikely to be without patients suffering from type 2 diabetes mellitus (T2DM) [2,3]. Furthermore, morbidity, mortality, and high healthcare costs are mainly caused by its long-term complications [2,4].

Numerous factors, including stress, inactivity, poor eating habits, obesity, genetics, aging, and inflammation, contribute to this disease; however, certain interventions, such as diet and weight loss plans, blood pressure and glucose monitoring, and exercise, can help manage the condition and its consequences [5].

Several hypoglycemic medications are commonly used for T2DM, including metformin, gliclazide, glucagon-like peptide-1 (GLP-1) analogues, and dipeptidyl peptidase-4 (DPP-4) inhibitors. In addition, hypoglycemic agents amplify insulin receptor sensitivity to food ingestion and reduce glucose levels to the normoglycemic range (plasma glucose 70-140 mg/dL or HbA1c 7%). Also, it is very necessary to balance the benefits of glycemic control with the adverse effects of drugs since current glucose-lowering medications do not cure diabetes [5,6].

Depending on the specific drug, these drugs can reduce HbA1c levels by 0.5%-1.5% in certain cases. Insulin treatment is common for type 1 diabetes mellitus (T1DM) patients and also common for T2DM patients when diet, exercise, weight loss, and oral hypoglycemic agents have not improved glycemic control [6,7].

Enteroendocrine cells secrete incretins, which control insulin postprandially. GLP-1 and glucose-dependent insulinotropic polypeptide (GIP) are two different types of incretins. DPP-4 is an enzyme that deactivates both of these incretins. GLP-1 can still function as an insulinotropic agent in T2DM patients, which activates GLP-1 receptors and improves insulin secretion, whereas GIP has no effect on the glucose-dependent secretion of insulin [8].

DPP-4 inhibitors are excellent blood glucose-dependent hypoglycemic agents to treat T2DM. These inhibitors increase endogenous GLP-1 levels by preventing DPP-4 enzymes from degrading incretins, which include GIP and GLP-1. GLP-1 contributes to the anti-hyperglycemic action by suppressing glucagon secretion from α-cells, inhibiting hepatic glucose synthesis, and stimulating insulin secretion from β-cells in a glucose-dependent manner. Furthermore, DPP-4 inhibitors maintain the bulk of the β-cell [9].

Apart from the widely recognized anti-hyperglycemia impact of the DPP-4 inhibitors, further new effects are gradually noticed, including cardiovascular protection and immune regulation, as well as an anti-inflammatory impact [10]. Some uncommon side effects of the DPP-4 inhibitor, including rheumatoid arthritis, hemolysis, leukopenia, and angioedema, have been reported recently as well [10-13].

As there are not many researches available either in the Qassim region or in other regions that aimed to assess the knowledge and concept of using DDP-4 inhibitors among family physicians, this research is about assessing several objectives for the use of DDP-4 inhibitors. This study, therefore, aimed to assess the prescribing practices and level of knowledge and general attitude of primary care physicians regarding DPP-4 inhibitors for the management of type 2 diabetes.

## Materials and methods

Study design and setting

Our study was an observational cross-sectional survey-based study design that was conducted from October 2024 to December 2024 in the public sector primary healthcare (PHC) centers in the Qassim region of Saudi Arabia.

Study population

The study population included all practicing primary care physicians in the Qassim region who were involved in the management of patients with T2DM.

Eligibility criteria

We included all the PHC physicians currently working in the Qassim region of either gender and all nationalities. Interns and physicians from other specialties were excluded. We also excluded those who refused to participate.

Sample size

Based on the estimated number of primary care physicians (359) practicing in the Qassim region, according to Ministry of Health statistics (www.moh.gov.sa/en), and an expected response rate of 80%, a sample size of 150 primary care physicians was required for this study.

Sampling procedure

A comprehensive list of all family physicians practicing in the Qassim region was obtained from the regional health authority. A random sampling approach was then used to select the PHC centers from the list available from the local administration. In the selected PHC centers, all the eligible physicians were invited to participate in the study.

Data collection tool

A self-administered questionnaire was developed based on previously validated instruments and aligned with the study objectives [14,15]. The questionnaire was used to collect sociodemographic information and to assess primary care physicians' knowledge, awareness, and prescribing practices regarding DPP-4 inhibitors for the management of type 2 diabetes.

Data collection procedure

The questionnaire was distributed to the selected primary care physicians either in person or electronically, based on their preferred mode of participation. Participants were provided with a detailed information sheet explaining the study's objectives and assuring the confidentiality of their responses.

Data analysis

The collected data were entered into an Excel sheet (Microsoft Corp., Redmond, WA, US), then organized and transferred to the Statistical Package for the Social Sciences (SPSS) version 25 (IBM Corp., Armonk, NY, US). Descriptive statistics were used to summarize the study variables. Continuous data were presented as means and standard deviations, while categorical variables were represented as frequencies and percentages. Inferential statistics, such as the independent sample t-test and ANOVA, were used to identify significant associations between physicians' characteristics and their knowledge regarding DPP-4 inhibitors. A p-value of less than 0.05 was considered statistically significant.

Ethical considerations

The study protocol was reviewed by the Qassim Regional Bioethics Committee (Approval number 607/46/3481). Participation in the study was entirely voluntary, and informed consent was obtained from all participants. The confidentiality of participants' responses was maintained throughout the study, and no personal identifiers were collected. The data were used for research purposes only.

## Results

This study included a total of 161 primary care physicians from the Qassim region. The average age of participants was 33.1 years, and the mean duration of clinical practice was 4.9 years. Men comprised 64.6% (n = 104) of the respondents, while women represented 35.4% (n = 57). In terms of specialty, family medicine physicians accounted for the majority (52.8%, n = 85), followed by family medicine trainees (24.8%, n = 40) and general practitioners (22.4%, n = 36). With respect to qualifications, 51.6% (n = 83) held board certifications, 46% (n = 74) had an MBBS degree, and a small minority (1.2%, n = 2) held a diploma (Table 1).

**Table 1:** Sociodemographic and professional characteristics of the participants SD: standard deviation; CME: continuing medical education; FM: family medicine

Variable	% (n)
Age	
Mean (SD)	33.1 (5.84)
Gender	
Male	64.6 (104)
Female	35.4 (57)
Years of experience	
Mean (SD)	4.9 (4.45)
Position	
General physician	22.4 (36)
Family physician	52.8 (85)
FM trainee	24.8 (40)
Highest qualification	
MBBS	46 (74)
Diploma	1.2 (2)
Board	51.6 (83)
Frequency of encounters with patients with type 2 diabetes	
Daily	78.9 (127)
Once in 2 days	19.3 (31)
Once in a week	1.9 (3)
Did you receive any CME on a novel class of drugs for type 2 diabetes management in the last year?	
Yes	26.1 (42)
No	73.9 (119)
Educational resources for dipeptidyl peptidase-4 (DPP-4) inhibitors are available in your institute.	
Yes	68.3 (110)
No	31.7 (51)

Most participants (78.9%) reported daily clinical interactions with patients diagnosed with T2DM. However, only 26.1% had attended continuing medical education (CME) sessions related to newer anti-diabetic therapies over the previous year. Moreover, 68.3% of respondents reported that information regarding DPP-4 inhibitors was readily accessible at their respective institutions (Table 1).

All participants (100%) were familiar with DPP-4 inhibitors. Nevertheless, certain knowledge deficiencies were noted. Only 60.9% correctly recognized DPP-4 inhibitors as a relatively recent advancement in T2DM therapy, and 59.5% acknowledged their potential role in lowering blood pressure. A majority (86.3%) understood that DPP-4 inhibitors are associated with a minimal risk of hypoglycemia. Awareness regarding the risk of pancreatitis linked to DPP-4 inhibitors was demonstrated by 76.4% of physicians, and 90.1% acknowledged their safe use in patients with chronic kidney disease (CKD). Additionally, 78.9% were aware that DPP-4 inhibitors could assist patients in achieving HbA1c targets within six months, while 77% were familiar with current Saudi and American Diabetes Association (ADA) guidelines governing their use (Table 2).

**Table 2:** Knowledge of PHC physicians about DPP-4 PHC: primary healthcare; DPP-4: dipeptidyl peptidase-4; CKD: chronic kidney disease

Question	% (n)
Are you aware of DPP-4 inhibitors?	
Yes	100 (161)
No	0 (0)
DPP-4 inhibitors are the latest addition in the management of type 2 diabetes mellitus.	
Yes	60.9 (98)
No	39.1 (63)
Administration of DPP-4 inhibitors does not cause hypoglycemia in type 2 diabetes patients.	
Yes	86.3 (139)
No	2.5 (4)
I am not sure	11.2 (18)
Using DPP-4 inhibitors can cause a reduction in blood pressure.	
Yes	59.5 (91)
No	19.3 (31)
I am not sure	24.2 (39)
DPP-4 inhibitor therapy can cause pancreatitis in diabetes patients.	
Yes	76.4 (123)
No	4.3 (7)
I am not sure	19.3 (31)
DPP-4 inhibitors are safe to prescribe to CKD patients.	
Yes	90.1 (145)
No	1.2 (2)
I am not sure	8.7 (14)

Physicians generally demonstrated a positive attitude toward DPP-4 inhibitors. Nearly 69.6% emphasized the necessity of obtaining informed consent before initiating therapy, and 93.8% reported a strong ethical commitment to discussing treatment risks and benefits with their patients. Almost all respondents (98.1%) indicated that they strive to tailor DPP-4 inhibitor selection based on individual patient profiles. Furthermore, 83.2% preferred prescribing DPP-4 inhibitors to patients with CKD, consistent with prevailing clinical guidelines (Table 3).

**Table 3:** Attitude of PHC physicians about DPP-4 PHC: primary healthcare; DPP-4: dipeptidyl peptidase-4; ADA: American Diabetes Association; CKD: chronic kidney disease

Question	% (n)
Is it essential to take consent from the patient before starting DPP-4 inhibitors?	
Yes	69.6 (112)
No	30.4 (49)
I feel a moral obligation to discuss the benefits and risk factors associated with DPP-4 inhibitor therapy.	
Yes	93.8 (151)
No	6.2 (10)
I make efforts to choose the most appropriate DPP-4 inhibitors specific for the patient.	
Yes	98.1 (158)
No	1.9 (3)
I chose DPP-4 inhibitors as the first choice for CKD patients.	
Yes	83.2 (134)
No	16.8 (27)
I recommend patients on DPP-4 inhibitor therapy to eat a small portion of meals and then wait 30 minutes before eating more.	
Yes	88.2 (142)
No	11.8 (19)
I prefer to encourage patients to self-monitor blood glucose a few times daily for a week or two after initiating the DPP-4 inhibitor therapy.	
Yes	95.7 (154)
No	4.3 (7)
Are you aware of the latest clinical Saudi/ADA guidelines for the use of DPP-4 inhibitors?	
Yes	77 (124)
No	23 (37)

Regarding prescribing behavior, a majority (72.7%) actively sought to update their knowledge on DPP-4 inhibitors through current literature. Reviewing patient history prior to prescribing was a routine practice for 96.9% of participants. Patient counseling on potential side effects was conducted by 98.1%, and nearly all participants (98.8%) avoided prescribing DPP-4 inhibitors during pregnancy or lactation. Additionally, 99.4% advised patients to promptly report symptoms suggestive of serious adverse effects, such as persistent abdominal pain (Table 4).

**Table 4:** Practice of PHC physicians about DPP-4 PHC: primary healthcare; DPP-4: dipeptidyl peptidase-4

Questions	% (n)
I devote time to reading literature for recent additions to the DPP-4 inhibitor class of anti-diabetic drugs.	
Yes	72.7 (117)
No	27.3 (44)
I always refer to the patient history before treating them with DPP-4 inhibitors.	
Yes	96.9 (156)
No	3.1 (5)
I inform patients taking DPP-4 inhibitor medication about the possible side effects such as nausea, diarrhea, and stomach pain.	
Yes	98.1 (158)
No	1.9 (3)
I avoid prescribing DPP-4 inhibitors to pregnant and nursing patients to prevent the complications of medications.	
Yes	98.8 (159)
No	1.2 (2)
I ask patients on DPP-4 inhibitor therapy to report immediately any unrelenting abdominal pain or hypersensitivity reactions.	
Yes	99.4 (160)
No	0.6 (1)
DPP-4 inhibitors reduce HbA1c to the target goal within 6 months of treatment.	
Yes	78.9 (127)
No	21.1 (34)

When exploring factors influencing knowledge levels, age and years of clinical experience were both positively correlated with higher knowledge scores (p < 0.001). Family physicians exhibited significantly better knowledge compared to general practitioners and trainees (p < 0.001), and board-certified physicians scored higher than those with only an MBBS or diploma qualification (p < 0.001). Furthermore, physicians with institutional access to educational materials scored significantly higher (p < 0.001). Although participation in CME activities showed a trend toward improved knowledge, the difference was not statistically significant (p = 0.150) (Table 5).

**Table 5:** Relationship between participant characteristics and knowledge scores SD: standard deviation; FM: family medicine; CME: continuing medical education

Variable	Knowledge score		p-value
	Mean (SD)	Test statistic	
Age			
Correlation coefficient (r)	0.357		<0.001
Gender			
Male	4.37 (1.10)	t, -1.605	0.101
Female	4.65 (0.94)		
Years of experience			
Correlation coefficient (r)	0.332		<0.001
Position			
General physician	3.86 (1.05)	F, 12.948	<0.001
Family physician	4.81 (0.78)		
FM trainee	4.28 (1.21)		
Highest qualification			
MBBS	4.04 (1.18)	F, 13.725	<0.001
Diploma	4.50 (0.71)		
Board	4.86 (.75)		
Frequency of encounters with patients with type 2 diabetes			
Daily	4.63 (0.93)	F, 10.411	<0.001
Once in 2 days	3.97 (1.11)		
Once in a week	2.67 (2.08)		
Did you receive any CME on a novel class of drugs for type 2 diabetes management in the last year?			
Yes	4.67 (0.82)	t, 1.675	0.097
No	4.39 (1.11)		
Educational resources on dipeptidyl peptidase-4 (DPP-4) inhibitors are available in your institute.			
Yes	4.65 (0.88)	t, 2.895	0.005
No	4.08 (1.26)		

## Discussion

In this study, we evaluated the knowledge, attitudes, and practices of primary care physicians in the Qassim region regarding the use of DPP-4 inhibitors in managing T2DM. Our findings showed that while the general awareness of DPP-4 inhibitors was high, several important knowledge and practice gaps remain. Also, all physicians surveyed were aware of DPP-4 inhibitors; only about 61% recognized them as relatively new agents for T2DM management, and approximately 59% were aware of their important role in reducing blood pressure. Similarly, knowledge about specific safety issues, such as the risk of pancreatitis or dose adjustment in CKD, was not optimal among all participants.

Comparable findings were reported in a study conducted in Riyadh by Alali et al. [14], where primary care physicians demonstrated high general awareness of novel anti-diabetic agents but lacked deeper knowledge regarding safety profiles and patient-specific considerations. Another Saudi study by Aljuaid et al. [15] found that only half of the surveyed physicians were familiar with newer diabetes treatments, including DPP-4 inhibitors. In contrast, international studies such as the CARMELINA trial showed that DPP-4 inhibitors, particularly linagliptin, are effective and safe for patients with CKD without the need for dose adjustment [16]. The knowledge gap in Saudi Arabia may reflect limited CME participation and insufficient integration of international guidelines into local practice.

The attitudes of physicians toward DPP-4 inhibitors were generally positive. Most participants felt a moral obligation to discuss the risks and benefits of therapy with patients, and many made efforts to choose the most appropriate drug based on patient needs. However, fewer physicians emphasized obtaining explicit patient consent before initiating DPP-4 therapy. Similar trends were seen in previous Saudi research, where physicians reported good intentions but did not consistently engage in shared decision-making [17]. Internationally, especially in Western countries, shared decision-making has become a standard part of diabetes care, leading to better treatment adherence and patient satisfaction [18]. These differences could stem from varying levels of emphasis on patient autonomy and communication skills in medical training programs.

When assessing actual practice patterns, our study found that while most physicians reviewed patient history and informed patients about side effects, only about 73% reported consistently updating their knowledge through recent literature. This finding mirrors that of Allyhiani et al. [17], who reported that Saudi physicians tend to rely on outdated prescribing habits, favoring older medications like sulfonylureas even when newer options are available. Globally, in systems where electronic health records and clinical decision support tools are widespread, physicians show higher adherence to evidence-based practices [19]. The lack of such systems in many Saudi primary care centers might explain part of the discrepancy.

Limited access to structured CME programs may contribute to the knowledge and practice gaps observed in our study. Similarly, in a study from Abha City, only 65.5% of PHC professionals attended CME courses, with 16.1% reporting them as unhelpful. Common barriers included a lack of time and a high workload. These findings suggest that while CME opportunities exist, their impact may be limited by accessibility and content relevance. CME programs could be more effective if they were customized to primary care physicians' needs and schedules [20].

To address these issues, several steps are necessary. CME activities focusing on new diabetes therapies should be mandatory and widely accessible, emphasizing not just awareness but also detailed clinical application, including dose adjustments in CKD and risk-benefit discussions. National guidelines should be regularly updated in alignment with international evidence and actively promoted among primary care providers. The use of electronic systems that deliver real-time updates and clinical reminders could also enhance physician adherence to updated protocols [21]. Additionally, training programs should incorporate communication and shared decision-making skills to promote more patient-centered care.

Our study is among the few attempts to understand the perspectives of primary care physicians about DPP-4 inhibitors. We developed the questionnaire based on existing literature. However, there are certain limitations that need to be considered while interpreting the results of this study. Firstly, we conducted this study in one city only, which may limit its generalizability. Secondly, there is a possibility of reporting bias where respondents may respond on social desirability. However, we assume it to have minimal effects on the validity of responses as data were completely anonymized and collected online. Finally, we included only one agent, i.e., DPP-4, while other alternatives are also available in the market and need similar investigation. This may reduce the scope of the research in terms of the number of drugs investigated. However, our approach does provide a focused and in-depth insight into DPP-4.

## Conclusions

Overall, while primary care physicians in the Qassim region exhibit good general awareness and positive attitudes toward DPP-4 inhibitors, significant gaps in knowledge and practice persist. Targeted educational interventions and better access to updated guidelines on newer therapies for T2DM are needed. Furthermore, system-wide reforms such as better CME opportunities and providing certification courses to enhance knowledge would improve patient outcomes. Further research is also needed to explore the availability of new drugs and the management of T2DM in the primary care setup.

## Appendices

**Table 6:** Questionnaire DPP-4: dipeptidyl peptidase-4; ADA: American Diabetes Association; CKD: chronic kidney disease; CME: continuing medical education; FM: family medicine

S. No.	Question	Response
Social and professional characteristics		
1	Age of the physician	-------years
2	Gender	1. Male
		2. Female
3	Years of experience as a physician	-------years
4	Position of the doctor	1. General physician
		2. Family physician
		3. FM trainee
5	Frequency of encounters with patients with type 2 diabetes	1. Daily
		2. Once in 2 days
		3. Once weekly
6	Did you receive any CME on a novel class of drugs for type 2 diabetes management in the last year?	1. Yes
		2. No
7	Educational resources on DPP-4 inhibitors are available in your institute.	1. Yes
		2. No
Knowledge of DPP-4		
1	Are you aware of DPP-4 inhibitors?	1. Yes
		2. No
		3. I am not sure
2	DPP-4 inhibitors are the latest addition in the management of type 2 diabetes mellitus.	1. Yes
		2. No
		3. I am not sure
3	Administration of DPP-4 inhibitors does not cause hypoglycemia in type 2 diabetes patients.	1. Yes
		2. No
		3. I am not sure
4	Using DPP-4 inhibitors can cause a reduction in blood pressure.	1. Yes
		2. No
		3. I am not sure
5	DPP-4 inhibitor therapy can cause pancreatitis in diabetes patients?	1. Yes
		2. No
		3. I am not sure
6	DPP-4 inhibitors are safe to prescribe to CKD patients.	1. Yes
		2. No
		3. I am not sure
Attitude questions		
7	Is it essential to take consent from patients before starting DPP-4 inhibitors?	1. Yes
		2. No
		3. I am not sure
8	I feel a moral obligation to discuss the benefits and risk factors associated with DPP-4 inhibitor therapy.	1. Yes
		2. No
		3. I am not sure
9	I make efforts to choose the most appropriate DPP-4 inhibitors specific for the patient.	1. Yes
		2. No
		3. I am not sure
10	I chose DPP-4 inhibitors to be my first choice for CKD patients.	1. Yes
		2. No
		3. I am not sure
11	I recommend patients on DPP-4 inhibitor therapy to eat a small portion of meals and then wait 30 minutes before eating more.	1. Yes
		2. No
		3. I am not sure
12	I prefer to encourage patients to self-monitor blood glucose a few times daily for a week or two after initiating the DPP-4 inhibitor therapy.	1. Yes
		2. No
		3. I am not sure
13	Aware of the latest clinical Saudi/ADA guidelines for the use of DPP-4 inhibitors.	1. Yes
		2. No
		3. I am not sure
Practice questions		
14	I devote time to reading literature for recent additions to the DPP-4 inhibitor class of anti-diabetic drugs.	1. Yes
		2. No
		3. I am not sure
15	I always refer to the patient history before treating them with DPP-4 inhibitors.	1. Yes
		2. No
		3. I am not sure
16	I inform patients taking DPP-4 inhibitor medication about the possible side effects such as nausea, diarrhea, and stomach pain.	1. Yes
		2. No
		3. I am not sure
17	I avoid prescribing DPP-4 inhibitors to pregnant and nursing patients to prevent the complications of medications.	1. Yes
		2. No
		3. I am not sure
18	I ask patients on DPP-4 inhibitor therapy to report immediately any unrelenting abdominal pain or hypersensitivity reactions.	1. Yes
		2. No
		3. I am not sure
